# Neurotrophic effects of perfluorocarbon emulsion gel: a pilot study

**DOI:** 10.1186/1749-7221-6-11

**Published:** 2011-11-23

**Authors:** Jonathan Isaacs, Ilvy Friebe, Satya Mallu, Keith Bachman

**Affiliations:** 1 Division of Hand Surgery, Department of Orthopaedic Surgery, Virginia Commonwealth University Health Systems, 1200 East Broad Street, P.O. Box 980153, Richmond, VA, USA

**Keywords:** Perfluorocarbons, Nerve Repair, Nerve Regeneration

## Abstract

**Background:**

Positive neurotrophic effects of hyperbaric oxygen treatment may be more easily achieved by applying a Perflourocarbon (PFC) emulsion gel to the repair site. PFCs are halogen substituted carbon oils with unique oxygen transport potentials that are capable of increasing oxygen availability in local tissues. The purpose of this study was to determine if the application of a PFC emulsion to a repaired nerve would improve recovery.

**Materials and methods:**

The left tibial nerve of 21 immature female Sprague-Dawley rats was transected, immediately repaired, and then circumferentially coated with PFC gel (Group A, n = 7), PFC-less gel (Group B, n = 7), or nothing (suture only, Group C, n = 7). At eight weeks post surgery, electrophysiological testing and histological and morphological analysis was performed.

**Results:**

No statistically significant differences between experimental groups were found for muscle size and weight, axon counts, or nerve conduction velocity. Group A had a significantly smaller G-ratio than Groups B and C (p < .0001).

**Conclusion:**

Overall results do not indicate a functional benefit associated with application of a PFC emulsion gel to rodent tibial nerve repairs. A positive effect on myelination was seen.

## Background

Despite significant advances in our understanding of nerve regeneration over the past six decades, achieving consistent satisfactory results following major nerve repair or reconstruction remains a challenge. Enhancement of the local biological environment towards a more neurosupportive environment is a common strategy aimed at improving nerve repair outcomes[1,2]. Strategies to increase tissue oxygenation at the repair site have received some attention in the past, though this has generally focused on hyperbaric oxygenation (HBO). Hyperbaric oxygenation treatment to improve nerve regeneration, involves emersion of the subject (patient or animal) in a pressurized and enriched oxygen environment for several hours at a time following the nerve repair. Results of this approach are mixed, but several reports suggest a positive effect[3-7].

Obvious problems with HBO include obtaining access to the expensive pressurized oxygenation chamber, the investment of prolonged unproductive periods of time within the chamber, and the possibly negative effects of fluctuating oxygen tensions associated with interval treatment schedules (i.e. high oxygenation levels will drop once the subject leaves the chamber). A less constrained approach for enhanced oxygen therapy may be possible through the use of perfluorocarbons. Perfluorocarbons are highly non-polar, biologically inert oils (first discovered during development of the atomic bomb), which possess unique gas transport potential. Perfluorocarbons (PFC) exhibit oxygen solubilities a factor of about 50× greater than that of water[8]. When applied around a nerve repair, PFCs may be able to provide local oxygen transport to damaged tissues without the disadvantages associated with HBO. The purpose of this study is to evaluate the effects of a PFC emulsion gel on functional nerve regeneration.

## Methods

The perfluorocarbon gel used was prepared using a proprietary PFC similar in properties and oxygen solubility to perfluorodecalin. The gel was prepared in a manner similar to that describe by Moore[9]. The isolated gel contained about 87 wt% of the PFC and was heat sterilized at 122°C for 2 hours before use.

Twenty-one immature (3 month old) Sprague-Dawley rats were used for study after obtaining necessary approval from our institution's animal review board in accordance with national guidelines. All animals were housed in a temperature and humidity controlled environment with 12:12 day-night cycle and were provided food and water ad libitum. The rats were divided into three groups (N = 7), A (repair with application of PFC emulsion gel), B (repair with application of PFC-less gel carrier only), and C (repair only). For all procedures, anesthesia was induced with 5% isoflurane in a closed chamber and maintained using 2-3% isoflurane via nose cone inhalation. Surgical manipulations of the left hind limb nerves were performed under sterile conditions. The sciatic, tibial, and peroneal nerves were exposed through a standard biceps femoris-semitendinosis muscle splitting approach. The tibial nerve was isolated and transected one centimeter distal to the bifurcation from the sciatic nerve. Under operating microscope magnification, the cut ends of the tibial nerve were immediately co-apted with two 10-0 nylon epineural sutures placed 180 degrees apart. The repair site for groups A and B were circumferentially coated with PFC emulsion gel (Group A) or gel carrier only (Group B). The wounds were closed with 4-0 nonabsorbent monofilament and the animals allowed to recover from anesthesia before being returned to their cages. Post-operative analgesia was consistently accomplished with subcutaneous administration of buprenorphine 0.5 mg/kg and acetaminophen 272 mg/100 cc added to the drinking water.

Final testing took place 8 weeks after the initial surgery. After the induction of general anesthesia, both the right and left sciatic nerves were exposed to allow testing on both the experimental and control hind limbs extremities. The sciatic nerve was isolated at the sciatic notch and proximal branches of the sciatic nerve transected to reduce muscle contraction and reduce interference. Two bipolar electrodes were placed on the isolated nerve with the stimulating electrode positioned at the proximal sciatic nerve and the recording bipolar electrode placed under the tibial nerve distally (with the interval space recorded for nerve conduction velocity calculation). Single square pulses of 0.02 msec duration were applied while gradually increasing the strength of the stimulus until a maximum compound action potential waveform was achieved. This maximum stimulus was applied three times to each nerve and latency, base to peak amplitude, and peak to peak amplitude measured and recorded using a PowerLab data acquisition system (ADInstruments, Inc., Colorado Springs, CO) and an Apple iBook laptop computer (Cupertino, California).

**Figure 1:**
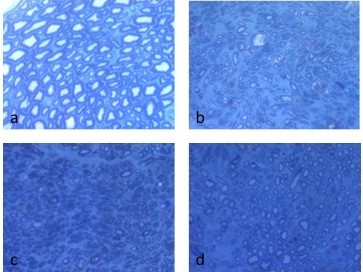
**Microscopic images in 40× magnification of (a) control nerve, (b) PFC gel to repair, (c) carrier only to repair and (d) repair only nerve 5 mm distal to repair site**.

Once nerve testing was complete, the tibial nerves and gastrocnemius muscles were harvested and fixed in 10% formalin solution. Nerve sections were obtained 5 mm proximal and 5 mm distal to the repair site and stained with toludine blue for histological analysis (Figure 1). Analysis consisted of averaged axon counts per 5 random high power fields (40× magnification) and G-ratio assessments using the same high power fields. Axon counts were performed manually using ImageJ 1.42 software (NIH website) and bias avoided by using the same five areas in each specimen. G-ratios were calculated by measuring the diameter of axons and dividing by the total diameter of that axon plus the surrounding myelin sheath. The gastrocnemius muscles were harvested from both hind limbs for diameter and weight measurements. The animals were euthanized with an intraperitoneal overdose of Euthasol (150 mg/kg).

Statistical analysis was performed using the ANOVA and Tukey's post test analysis, comparing the individual experimental groups versus all control groups, as well as the experimental groups to each other.

### Power analysis

Power analysis sample size determination was based on standard deviations and reported differences noted in similar studies and using GraphPad StatMate software (GraphPad Software, Inc., San Diego, CA). The study by Eguiluz-Ordonez et al. used a similar rodent sciatic nerve model but looked at the effect of post repair exposure to HBO. They found a statistically significant difference in motor latency (mean difference .46 ms, average Standard Deviation 0.34) with 10 rats in each group[6]. To achieve 90% power, seven rats in each group would demonstrate this difference if a similar standard deviation is expected.

## Results

### Muscle Weight and Diameter

For all experimental limbs, the gastrocnemius muscle measurements demonstrated statistically significant lighter weights and smaller diameters (p < 0.0001) when compared with control limbs. The average weight and diameter of the experimentally manipulated muscles were 59% and 73% of the control side, respectively. There was no statistically significant difference in muscle size or weight between the three experimental groups (Table 1).

**Table 1:** Results of testing for all three experimental groups and control limbs

	Exp -A (PFC)		Exp -B (Gel)		Exp -C (repair only)		Control	
	mean	SD	mean	SD	mean	SD	mean	SD
**Axon Count**	175.0	37.66	184.9	16.56	189.3	31.66	189.7	28.45
**Muscle Diameter (mm)**	6.19	1.06	6.82	0.38	7.76	1.09	9.45	0.54
**G-ratio**	0.6136*	0.1322	0.6863	0.0808	0.6858	0.0974	0.6465**	0.0749
**Muscle Weight (g)**	1.119	0.1299	1.152	0.1002	1.241	0.1070	1.998**	0.2112
**Motor Latency (msec) (N to N)**	2.459	0.3596	2.671	0.3974	2.477	0.3406	1.944**	0.3942
**Motor Latency (msec) (N-M)**	3.030	0.4727	3.094	0.9046	2.646	0.6688	2.362**	0.3689
**Amplitude (μV)** **Base to Peak**	1833	1292	2788	3028	2675	1255	5983**	3908
**Amplitude (μV)** **Peak to Peak**	3084	2031	3611	3287	4070	2211	8980**	6465

* statistically significant difference to Exp-B and Exp-C

** statistically significant to all Exp (A, B, C) groups

### Axon Count

There was no statistically significant difference in axon counts (p = 0.09) per averaged high power field between the experimental and control groups, nor within the experimental groups (Table 1).

### Nerve Conduction Velocity

There was a significant increase in tibial nerve motor latency (p < 0.05), a decrease in tibial nerve amplitude (p < 0.0001) in all three experimental groups compared to the control nerves but no differences between the experimental groups (Table 1).

### Histomorphometry

There was a significantly increased G-ratio in the experimental groups compared to the control groups (p < 0.05). Among the experimental groups, Group A (PFC) had a significantly smaller G-ratio than Groups B and C (p < 0.0001) (Table 1).

## Discussion

Perfluorocarbons are unique compounds created when hydrocarbon chains undergo a hydrogen substitution with fluorine molecules. The subsequent oils are stable, inert, and have low surface tensions. Because they are highly non-polar and extremely hydrophobic, they will not bind to proteins and are usually lipophilic. Used in a variety of industrial and medical applications, PFCs possess impressively high oxygen (and other gas) solubility potentials which has spurred interest in a potential role as a "blood substitute". Oxygen molecules do not bind to the perfluorcarbon molecules (like they do to hemoglobin), but rather are trapped in between the molecules. This dissolved oxygen can be made available to surrounding tissues in certain circumstances [8]. When applied directly to a tissue bed, PFC gel has also been shown to improve wound healing[10]. We have hypothesized that application of a PFC emulsion gel around a repaired nerve might be expected to increase oxygen availability at the repair site and subsequently result in improved nerve regeneration.

Possible beneficial mechanisms of improved oxygenation on nerve regeneration include promotion of survival of marginal tissue, reduced edema and improved microcirculation, and up-regulation of growth factors[11]. Additionally, low oxygen tension has been correlated with scar tissue formation[12,13]. Scar tissue can block regenerating axons acutely or, more chronically, can cause secondary compression resulting in "strangulation" of the nerve and traction neuritis. Both can inhibit nerve function and may contribute to chronic neuropathic pain.

Increased oxygen tissue saturation as seen with HBO treatment has demonstrated potential benefits for nerve regeneration in both experimental and clinical settings. Zamboni et al. demonstrated improved functional recovery (walking track analysis) and decreased perineural scarring in rats treated with hyperbaric oxygen (HBO) (2.5 ATA/90 min/BID/7 days) following sciatic nerve injury (with nerves "stripped" of extrinsic blood supply)[3]. Using electron and light microscopy, Bradshaw et al. found more complete recovery of crushed rabbit sciatic nerves following HBO treatment[4]. Likewise, Haapaniemi et al. demonstrated improved axonal regeneration into nerve grafts when experimental rats received HBO treatment immediately following repair[5]. Eguiluz et al., while unable to demonstrate histological improvement with HBO treatment, noted improved functional and electrophysiological recovery in a rat sciatic nerve injury[6]. From a clinical perspective, Zhao reported on the beneficial effects of HBO combined with prompt surgical repair of nerve injuries[7].

Similar benefits as seen with HBO treatment were not demonstrated in our study. Morphological and electrophysiological data did not demonstrate any significant positive or negative effect when compared to either the carrier gel group or to the repair only group. A lower G-ratio, however, indicating a higher myelin to axon diameter ratio, was found in the PFC group. Similar positive effects on myelination have been seen with HBO[4,14]. Though this may represent faster axon regeneration and maturation (as has also been associated with HBO treatment in other rodent nerve repair models [5,15]), our study design did not specifically evaluate this variable and the exact significance or etiology of this finding is not known. Our evaluation period of only 8 weeks after nerve repair, however, was chosen specifically to identify this type of subtle difference in nerve regeneration between groups as longer recovery periods in similar rodent models have been recently associated with a "blow though effect" --eventually resulting in robust axonal regeneration regardless of treatment variables[16].

Our failure to demonstrate definite neurotrophic benefits with the PFC emulsion gel, however, has several possible explanations. First, although oxygen delivery to the nerve should have improved theoretically with PFC application, this was unable to be verified and without this effect, the emulsion gel would have no beneficial properties. Though this seems like a grave oversight in the study design, it must be pointed out that accurate measurements of oxygen tension at an in vivo nerve repair site (such as in this model) would be impossible. Furthermore, none of the already referenced studies regarding the effects of HBO on nerve healing and regeneration confirmed increased perineural or neural oxygen tension. Second, oxygenation (or perfusion) of the neural tissue may not have been an issue with this straightforward repair model[17] and the potential benefits of improving oxygenation may only be seen with ischemic tissue. A "rescue effect" has been demonstrated in experimental ischemic nerve tissue treated with HBO[18]. Other studies, however, fail to support this explanation. No functional or histological effect was seen with HBO treatment in an entubulated, acellular, or "hypoxic" rodent nerve repair model[15,19-21].

The final possible explanation for the lack of neurotrophic benefit seen in our study is that the results of previous HBO studies are overstated and that increased oxygenation at the nerve repair site may have no effect or may even be potentially harmful. In contrast to the supportive studies already cited, other studies have failed to demonstrate any benefit of HBO on nerve regeneration[19,22,23]. Potential counter productive effects of HBO could include decreased inflammatory cell responses[24] (which might affect macrophages necessary to clear debris from the endoneural tubes which is essential for axon regneration), though no specific macrophage effect has been demonstrated with HBO[21], and we did not evaluate macrophages in our study. Additionally, HBO has been demonstrated to increase nitrous oxide (NO) production[25]. Nitrous oxide may actually inhibit nerve regeneration and even be neurotoxic[26-28].

The above theories and apparently contradictory experimental study results may not be in complete conflict. It is likely that the situation is more complicated than presented and that there may be a "sweet spot" for oxygen levels. Too little or too much oxygen may both be detrimental to nerve regeneration. In this study, PFC emulsion gel when applied directly to a primary rodent tibial nerve repair increased myelination of regenerating axons but did not demonstrate any net positive neurotrophic effect. Further study to confirm local tissue effects of PFC gel application as well as a dose response curve would be appropriate before any final conclusions are made.

## Conclusion

Although increased axonal myelination was noted, this was not associated with functional benefit following the application of a PFC emulsion gel to a tibial nerve repair in a rodent model.

## Competing interests

The authors declare that they have no competing interests.

## Authors' contributions

JI designed the project and wrote the manuscript. SM oversaw the rodent surgeries and was assisted by either IF or KB. GH and MQ developed and prepared the PFC emulsion gel. All authors read and approved the final manuscript.

## Authors Information

Co-author's contact information:

Ilvy Friebe, M.D.

1200 East Broad Street

P.O. Box 980153

Richmond, VA 23298

ifriebe@mcvh-vcu.edu

Phone: 804-828-3815

Fax: 804-828-4762

Satya Mallu, M.D.

1200 East Broad Street

P.O. Box 980153

Richmond, VA 23298

smallu@mcvh-vcu.edu

Phone: 804-828-3815

Fax: 804-828-4762

Keith Bachman, B.S.

3106 Ludlow Road

Shaker Heights, OH 44120

Krb3b.uva@gmail.com

Phone: 804-432-0025
